# Anti-proliferative and anti-migratory properties of coffee diterpenes kahweol acetate and cafestol in human renal cancer cells

**DOI:** 10.1038/s41598-020-80302-4

**Published:** 2021-01-12

**Authors:** Tomoyuki Makino, Kouji Izumi, Kaoru Hiratsuka, Hiroshi Kano, Takashi Shimada, Taito Nakano, Suguru Kadomoto, Renato Naito, Hiroaki Iwamoto, Hiroshi Yaegashi, Kazuyoshi Shigehara, Yoshifumi Kadono, Hiroki Nakata, Yohei Saito, Kyoko Nakagawa-Goto, Norihiko Sakai, Yasunori Iwata, Takashi Wada, Atsushi Mizokami

**Affiliations:** 1 Department of Integrative Cancer Therapy and Urology, Kanazawa University Graduate School of Medical Science, 13-1 Takara-machi, Kanazawa, Ishikawa 920-8640 Japan; 2 Department of Histology and Cell Biology, Kanazawa University Graduate School of Medical Science, Kanazawa, Japan; 3 School of Pharmaceutical Sciences, College of Medical, Pharmaceutical and Health Science, Kanazawa University, Kanazawa, Japan; 4 Department of Nephrology and Laboratory Medicine, Kanazawa University, Kanazawa, Japan

**Keywords:** Renal cancer, Cell growth, Cell migration, Drug development

## Abstract

Despite improvements in systemic therapy options for renal cancer, it remains one of the most drug-resistant malignancies. Interestingly, reports have shown that kahweol and cafestol, natural diterpenes extracted from coffee beans, exhibit anti-cancer activity. However, the multiple potential pharmacological actions of both have yet to be fully understood. This study therefore investigated the effects of kahweol acetate and cafestol on human renal cancer ACHN and Caki-1 cells. Accordingly, the combination of kahweol acetate and cafestol administration synergistically inhibited cell proliferation and migration by inducing apoptosis and inhibiting epithelial–mesenchymal transition. Mechanistic dissection revealed that kahweol acetate and cafestol inhibited Akt and ERK phosphorylation. Moreover, kahweol acetate and cafestol downregulated the expression of not only C–C chemokine receptors 2, 5, and 6 but also programmed death-ligand 1, indicating their effects on the tumor microenvironment. Thus, kahweol acetate and cafestol may be novel therapeutic candidates for renal cancer considering that they exert multiple pharmacological effects.

## Introduction

Renal cancer represents the 6th and 10th most frequently diagnosed malignancy among men and women, accounting for 5% and 3% of all oncological diagnoses in the United States, respectively^1^. Increased tumor size and metastasis have been the major causes of renal cancer-related death^2,3^. Despite advancements in molecular-targeted therapies and immunotherapies, such as tyrosine kinase inhibitors targeting vascular endothelial growth factor (VEGF) and immune checkpoint inhibitors^4–8^, advanced renal cancer remains among the most drug-resistant malignancies. As such, novel therapeutic strategies that improve prognosis of patients with renal cancer are urgently needed.

Reports have suggested that natural products may serve as substitutes for anti-cancer agents with novel biochemical mechanisms that inhibit cell proliferation, modulate cell differentiation, and induce apoptosis^9^. We had previously reported, for the first time, that kahweol acetate and cafestol, natural diterpenes extracted from coffee beans, synergistically inhibit prostate cancer cell progression in vitro and in vivo^10^. Kahweol acetate and cafestol have a wide variety of bioactive properties, including anti-inflammation, anti-angiogenesis, and anti-tumorigenic properties and it is estimated that three or four cups of coffee reach bioactive serum concentration of kahweol and cafestol^11^. Therefore, the present study sought to examine the anti-cancer activity of kahweol acetate and cafestol in renal cancer cells.

## Results

### Suppression of human renal cancer cell proliferation following kahweol acetate and cafestol treatment

**Figure 1:**
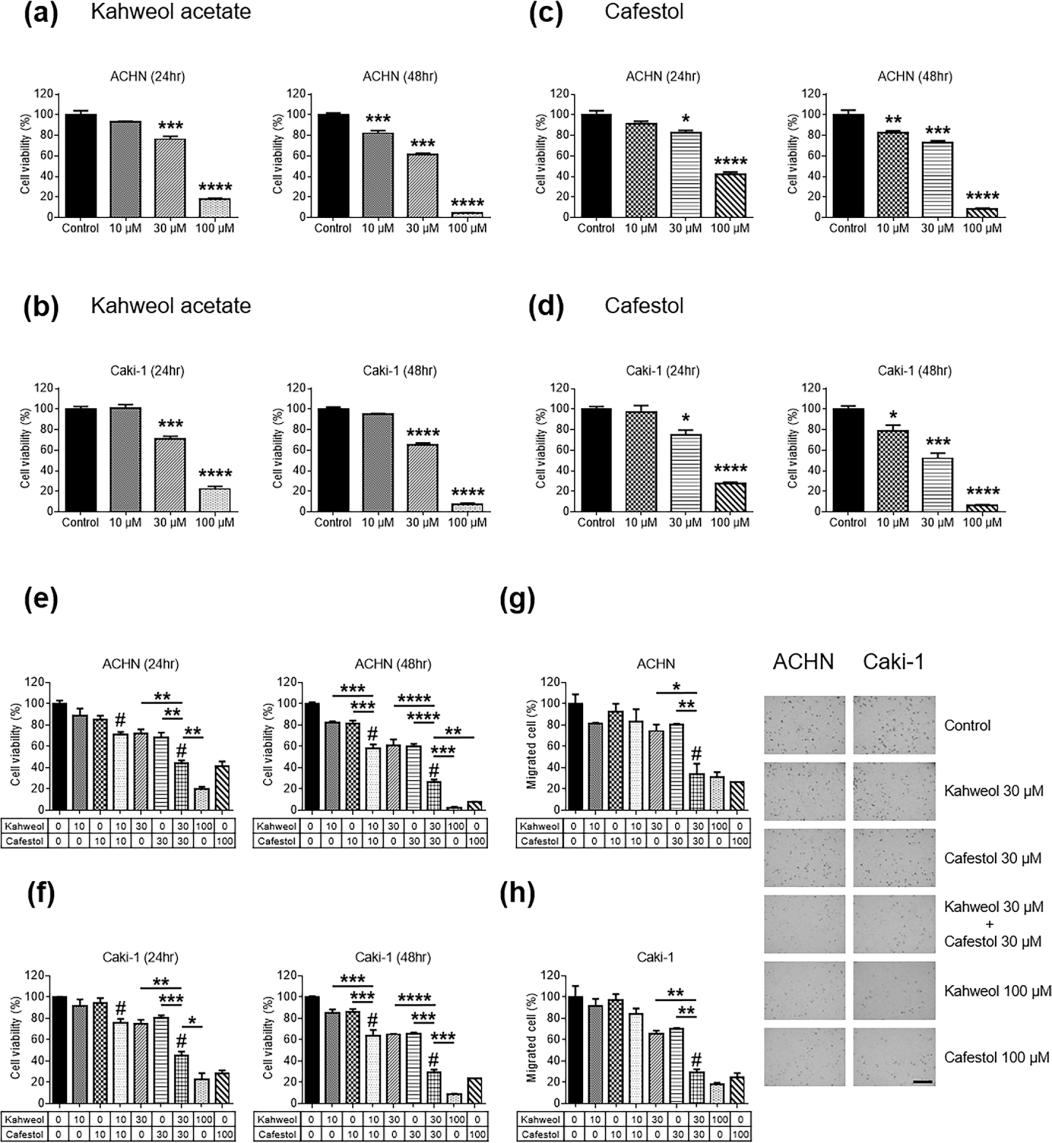
Anti-proliferative and migration effects of kahweol acetate and cafestol on renal cancer cells. (**a**–**d**) ACHN (**a**,**c**) and Caki-1 (**b**,**d**) cells were seeded in 12-well plates (5 × 10^4^ cells/well) with RPMI containing 10% FBS. Each cell was treated with or without pre-determined concentrations of kahweol acetate and cafestol for 24 and 48 h. (**e**,**f**) Anti-proliferative effects of the combination of kahweol acetate and cafestol on ACHN (**e**) and Caki-1 (**f**) cells for 24 and 48 h. (**g**,**h**) Anti-migration effect of the combination of kahweol acetate and cafestol on ACHN (**g**) and Caki-1 (**h**) cells for 12 h. Bar = 500 µm. All experiments were performed in triplicate. Data are presented as means ± standard error of the mean. *p < 0.05; **p < 0.01; ***p < 0.001; ****p < 0.0001. ^#^Synergistic effects were observed.

Considering our previous findings suggesting that kahweol acetate and cafestol had strong anti-proliferative effect in prostate cancer cells^10^, we determined whether kahweol acetate and cafestol could suppress cell proliferation of human renal cancer cells. Accordingly, kahweol acetate treatment significantly inhibited ACHN and Caki-1 proliferation after 24 and 48 h in a dose-dependent manner, respectively (Fig. 1a,b). Compared to control, ACHN and Caki-1 proliferation was around 40% and more than 90% lower 48 h after 30 and 100 µM of kahweol acetate treatment, respectively. Similarly, cafestol treatment significantly inhibited ACHN and Caki-1 cell proliferation after 24 and 48 h in a dose-dependent manner, respectively (Fig. 1c,d). Compared to control, ACHN and Caki-1 cells proliferation was 30–50% and more than 90% lower 48 h after 30 and 100 µM of cafestol treatment, respectively. To examine whether kahweol acetate and cafestol affect normal kidney cells, proliferation assay of proximal tubular cells from normal adult human kidney (HK-2) was performed. Since the combination of 30 µM kahweol acetate and 30 µM cafestol did not affect viability of HK-2 cells at all, 30 µM of kahweol acetate and cafestol was thought as non-cytotoxic concentrations of kahweol acetate and cafestol (Supplementary Figure S1). These results indicate that medium concentrations (10–30 µM) of kahweol acetate and cafestol exert an anti-proliferative effect on renal cancer cells without impairment of normal kidney cells.

### Synergistic inhibition of human renal cancer cell proliferation and migration following combined kahweol acetate and cafestol treatment

Given that kahweol acetate and cafestol are both derived from coffee, these diterpenes can be ingested concurrently when drinking coffee^11,12^. In fact, we had also reported that the combination of these diterpenes synergistically affected prostate cancer cell viability^10^. The anti-cancer effects of combined kahweol acetate and cafestol treatment on human renal cancer cell proliferation and migration was evaluated by calculating the combination index (CI)^13^. Accordingly, the combination of 30 µM kahweol acetate and 30 µM cafestol promoted greater inhibition of ACHN and Caki-1 proliferation after 24 and 48 h compared to 30 µM kahweol acetate or 30 µM cafestol alone (CI < 1; synergism) (Fig. 1e,f). Even low-dose combination treatment (i.e., 10 µM kahweol acetate and 10 µM cafestol for 24 and 48 h) promoted inhibition of cell proliferation comparable to 30 µM of each diterpene treatment alone (CI < 1). Given studies showing that diterpenes had multifunctional anti-cancer effects^11,12,14^, cell migration assays were also conducted to verify the effects of kahweol acetate and cafestol on ACHN and Caki-1 cells. Similar to results of proliferation assays, the combination of 30 µM kahweol acetate and 30 µM cafestol promoted significantly greater inhibition of cell migration compared to 30 µM kahweol acetate or 30 µM cafestol alone (CI < 1). In addition, we performed wound healing assay to further confirm above results from transwell migration assay. The combination of 30 µM kahweol acetate and 30 µM cafestol promoted significantly greater inhibition of cell migration compared to 30 µM kahweol acetate or 30 µM cafestol alone (Supplementary Figure S2). Importantly, the combination of 30 µM of diterpenes showed comparable effects to 100 µM of each diterpene alone (Fig. 1g,h).

### Apoptosis and downregulation of anti-apoptotic proteins in human renal cancer cells following kahweol acetate and cafestol treatment

**Figure 2:**
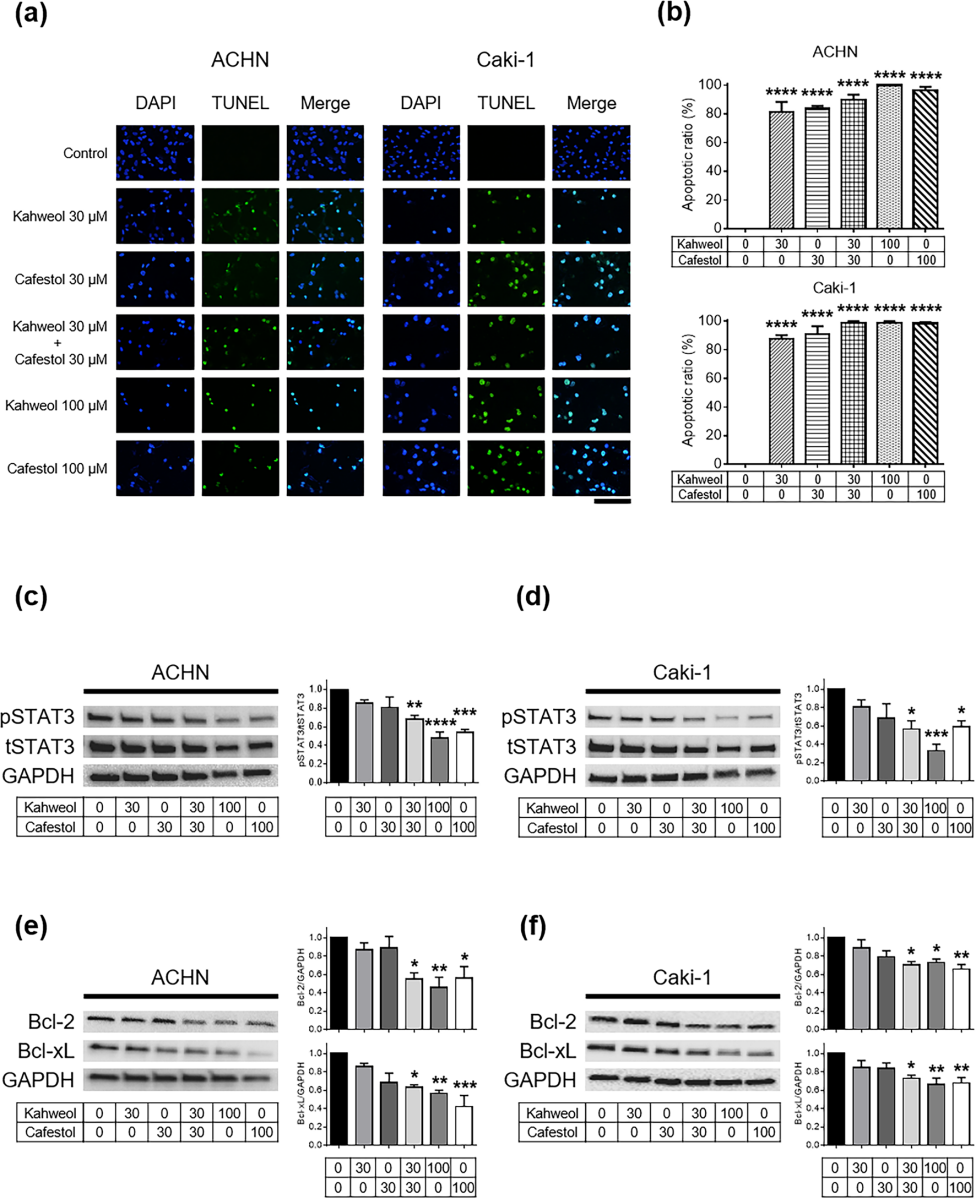
Kahweol acetate and cafestol treatment induced apoptosis by downregulating anti-apoptotic proteins in renal cancer cells. (**a**,**b**) TdT-mediated dUTP-biotin nick end labeling (TUNEL) assay (**a**) and statistics (**b**) showing apoptosis of ACHN or Caki-1 cells under kahweol acetate and cafestol treatment. ACHN and Caki-1 cells (3 × 10^4^ cells) were exposed to predetermined concentrations of kahweol acetate and cafestol for 12 h on sterile slide coverslips, after which TUNEL assays were performed. All experiments were performed in triplicate. Data are presented as means ± standard error of the mean (SEM). Bar = 50 µm. (**c**–**f**) Protein levels of apoptosis-associated genes, p/tSTAT3 in ACHN (**c**) and Caki-1 (**d**) cells, Bcl-2/Bcl-xL in ACHN (**e**) and Caki-1 (**f**) cells, treated for 24 h were measured using Western blot analysis and quantitatively analyzed using densitometry with ImageJ software as shown by bar graphs. Equal amounts of cell lysates (10 µg) were subjected to electrophoresis. Data are presented means ± SEM (n = 4). *p < 0.05; **p < 0.01; ***p < 0.001; ****p < 0.0001.

TdT-mediated dUTP-biotin nick end labeling (TUNEL) assays of ACHN and Caki-1 cells were performed to assess whether kahweol acetate and cafestol could induce apoptosis. Consistent with the results of proliferation assays, kahweol acetate and cafestol strongly induced apoptosis, even with 30 µM of each diterpene alone (Fig. 2a,b). Next, western blot analyses were performed to determine changes in key proteins involved in cancer cell proliferation, including apoptosis-related proteins. Accordingly, the combination of 30 µM kahweol acetate and 30 µM cafestol hampered STAT3 activation (Fig. 2c,d). Moreover, the combination of 30 µM kahweol acetate and 30 µM cafestol promoted significantly lower expression of Bcl-2 and Bcl-xL, downstream of STAT3, compared to untreated cells (Fig. 2e,f). In addition, Bcl-2-associated X protein (Bax) increased by kahweol acetate and cafestol (Supplementary Figure S3). We further checked caspase-related apoptosis proteins to examine whether apoptosis induced by kahweol acetate and cafestol was caspase-dependent or not, there were no fragmentation of caspase-3 and PARP observed regardless of the concentration of kahweol acetate and cafestol (Supplementary Figure S4). The combination of 30 µM diterpenes inhibited the aforementioned apoptosis-related proteins to an extent comparable to high concentrations (100 µM) of each diterpene alone.

### Downregulation of epithelial–mesenchymal transition-related proteins following kahweol acetate and cafestol treatment

**Figure 3:**
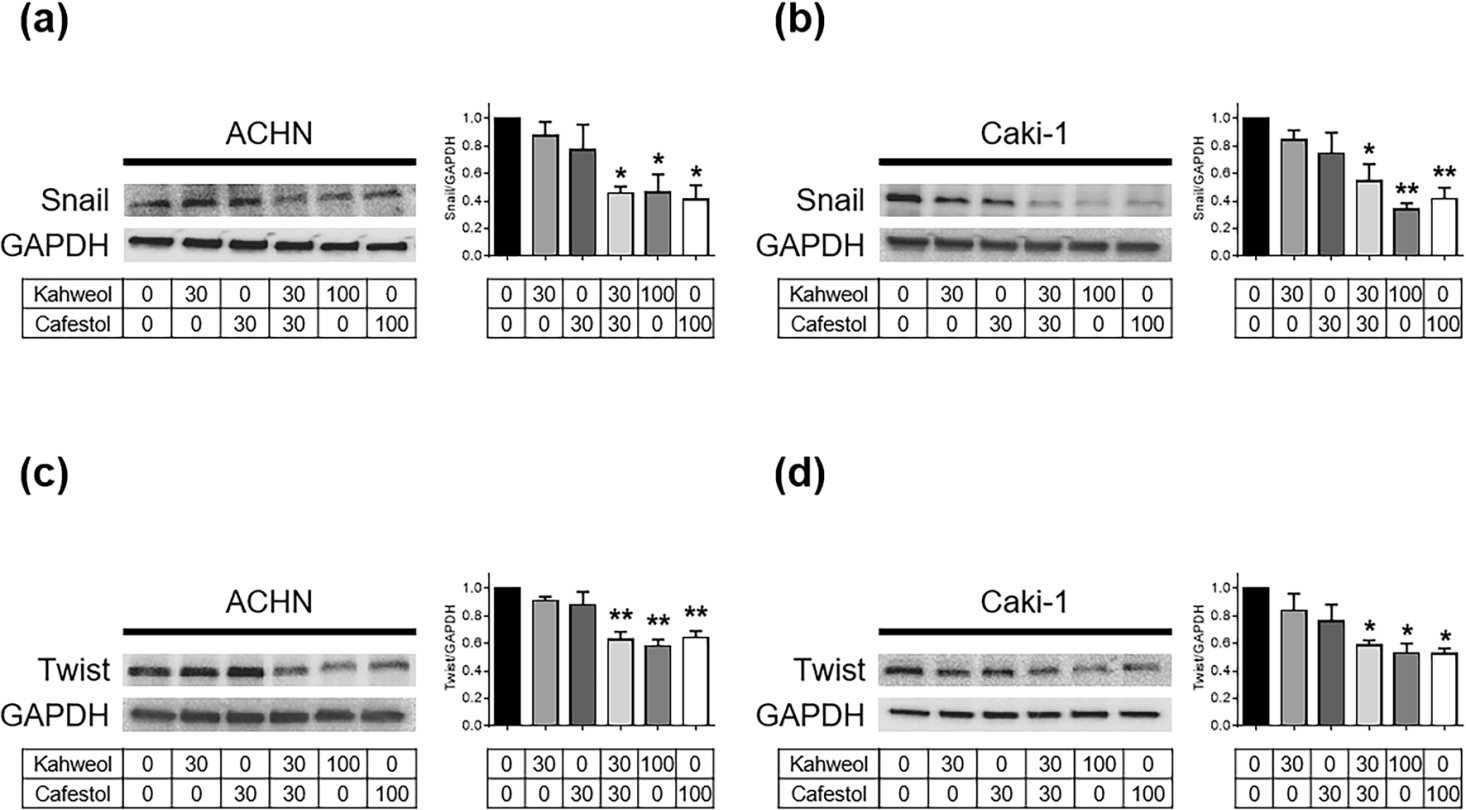
Kahweol acetate and cafestol treatment promoted downregulation of epithelial–mesenchymal transition (EMT)-related proteins in renal cancer cells. (**a**–**d**), Protein levels of EMT-associated genes, Snail in ACHN (**a**) and Caki-1 (**b**) cells, Twist in ACHN (**c**) and Caki-1 (**d**) cells, treated for 24 h were measured using Western blot analysis and quantitatively analyzed using densitometry with ImageJ software as shown by bar graphs. Soluble cell lysates (30 µg in Snail, 10 µg in Twist) were subjected to electrophoresis. Data were presented as means ± standard error of the mean (n = 4). *p < 0.05; **p < 0.01.

Western blot analyses of epithelial–mesenchymal transition (EMT)-related proteins were performed to trace the mechanism whereby cell migration was inhibited. Accordingly, the combination of 30 µM kahweol acetate and 30 µM cafestol, as well as high concentrations (100 µM) of each diterpene alone, clearly reduced Snail expression in ACHN and Caki-1 cells (Fig. 3a,b). The combination of 30 µM kahweol acetate and 30 µM cafestol, as well as high concentrations (100 µM) of each diterpene alone, also clearly reduced expression of Twist, which had been reported to interact with Snail^15^, in ACHN and Caki-1 cells (Fig. 3c,d). Among other EMT-related proteins we examined, although E-cadherin and Slug did not express at all regardless of the concentration of kahweol acetate and cafestol, N-cadherin and β-catenin showed the tendency to decrease by kahweol acetate and cafestol in ACHN and Caki-1 cells (Supplementary Figure S5). The aforementioned results indicated that kahweol acetate and cafestol decreased migration ability of human renal cancer cells through the inhibition of EMT.

### Downregulation of Akt and ERK signaling following kahweol acetate and cafestol treatment

**Figure 4:**
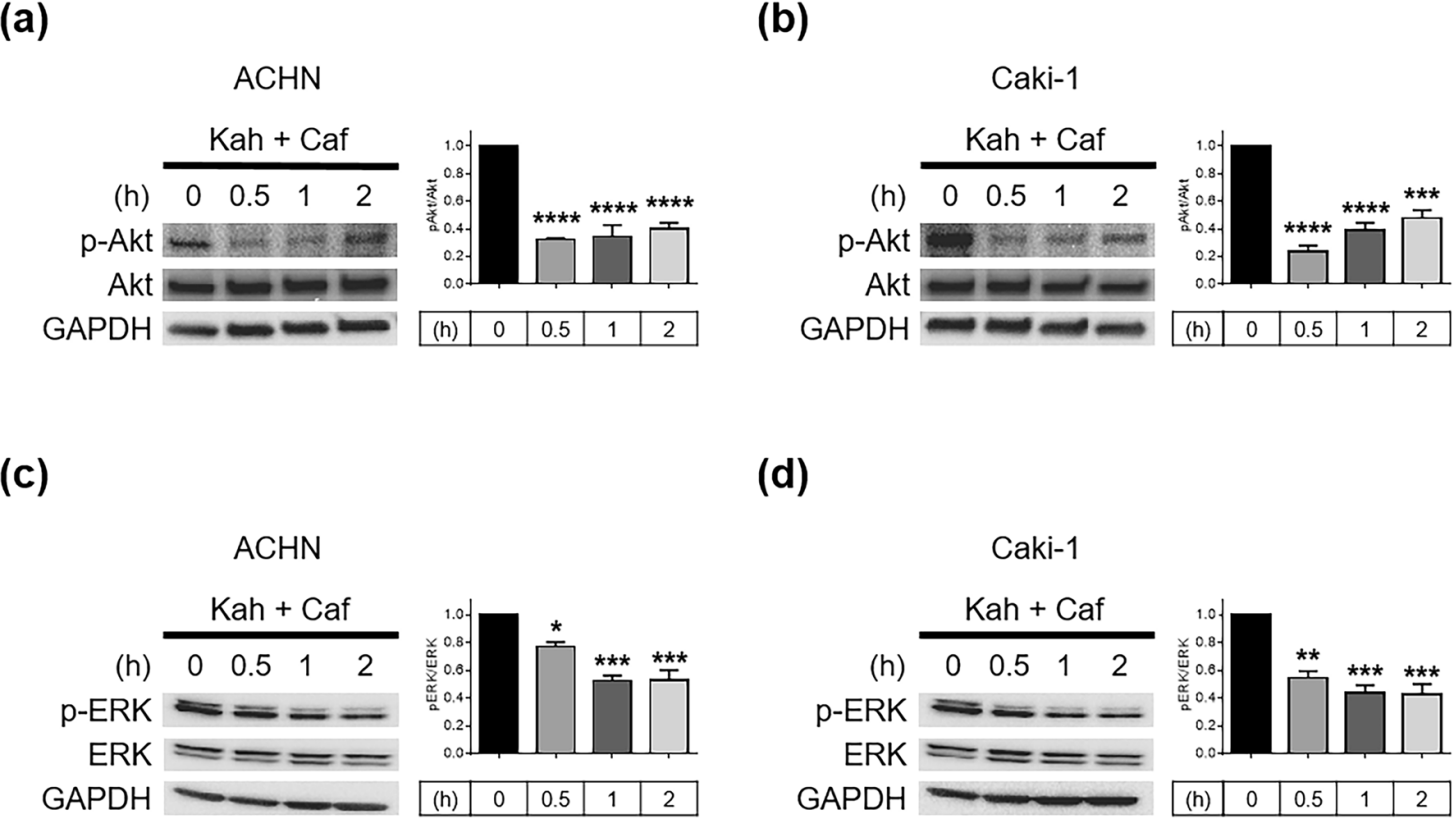
The combination of 30 µM kahweol acetate and 30 µM cafestol inhibited Akt and ERK signaling in renal cancer cells. (**a**–**d**) Phosphorylation of Akt in ACHN (**a**) and Caki-1 (**b**) cells, phosphorylation of ERK in ACHN (**c**) and Caki-1 (**d**) cells, at the indicated times after combination treatment were assessed using Western blot analysis and quantitatively analyzed using densitometry with ImageJ software as shown by bar graphs. Equal amounts of cell lysates (10 µg) were subjected to electrophoresis. Data are presented as means ± standard error of the mean (n = 3). *p < 0.05; **p < 0.01; ***p < 0.001; ****p < 0.0001.

Studies have reported Akt and ERK as factors strongly associated with the acceleration of renal cancer cell aggressiveness in terms of both growth and metastasis^16,17^. Akt and ERK, which promote EMT, are activated by STAT3 through downstream signaling^18,19^. Accordingly, the combination of 30 µM kahweol acetate and 30 µM cafestol rapidly inhibited Akt (Fig. 4a, b) and ERK phosphorylation (Fig. 4c,d) in ACHN and Caki-1 cells.

### Inhibition of immune signaling proteins after kahweol acetate and cafestol treatment

**Figure 5:**
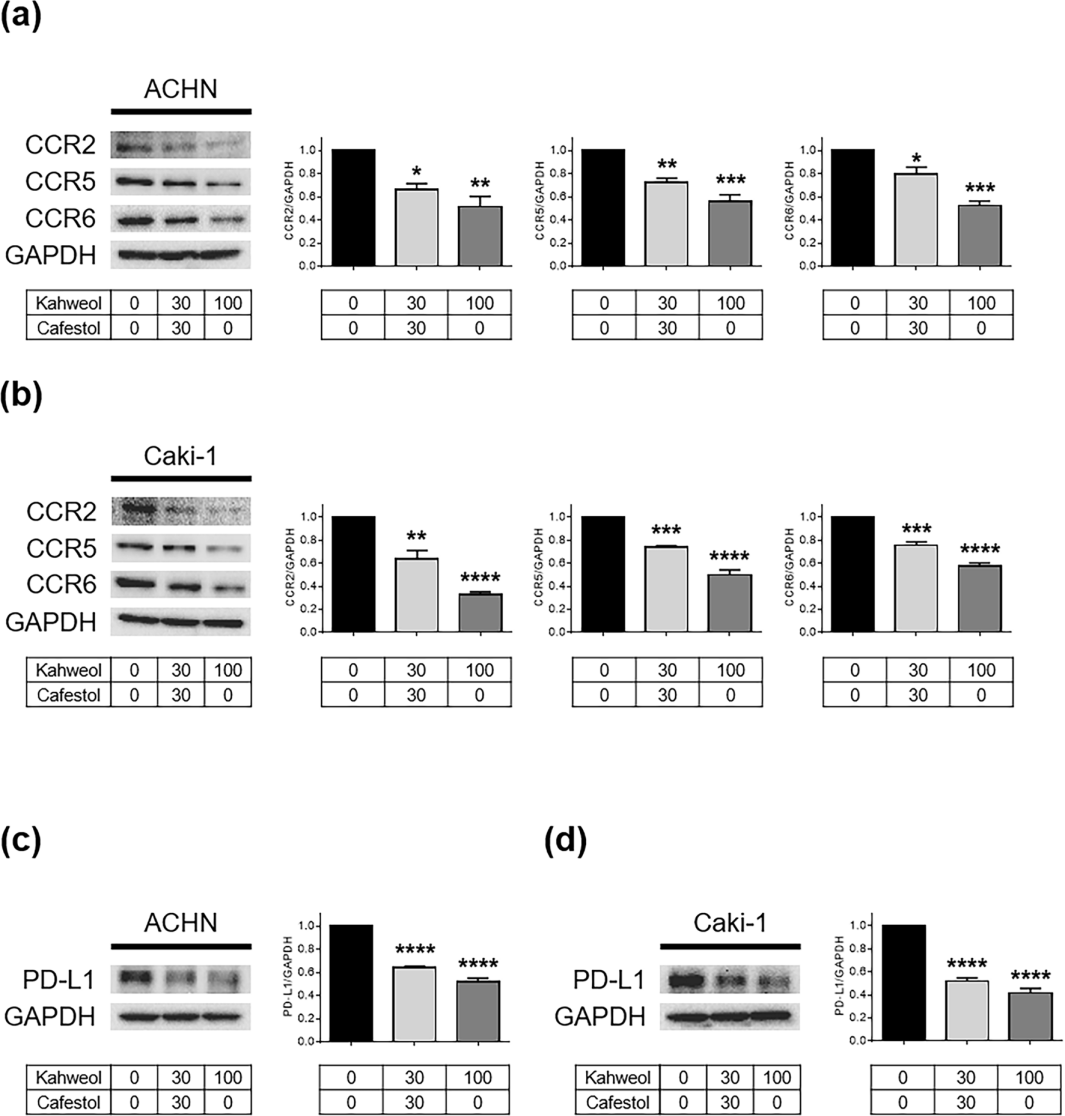
Kahweol acetate and cafestol treatment promoted the downregulation of immune signaling molecules in renal cancer cells. (**a**–**d**) Protein levels of C–C chemokine receptors (CCRs; CCR2/5/6) in ACHN (**a**) and Caki-1 (**b**) cells, and programmed death-ligand 1 (PD-L1) in ACHN (**c**) and Caki-1 (**d**) cells treated for 24 h were measured using Western blot analysis and quantitatively analyzed using densitometry with ImageJ software as shown by bar graphs. The soluble cell lysates (10 µg in CCR2/5 and PD-L1, 20 µg in CCR6) were subjected to electrophoresis. Data were presented as means ± standard error of the mean (n = 3). *p < 0.05; **p < 0.01; ***p < 0.001; ****p < 0.0001.

Cancer progression can be regulated by not only internal cancer cell signals but also external factors. Some immune cells that infiltrate into cancer tissues promote cancer progression by secreting chemokines that often activate cancer cells^20,21^. We thus investigated the influence of kahweol acetate and cafestol on C–C chemokine receptors (CCR2, 5, and 6) of representative chemokines activating renal cancer cells^22–26^. Accordingly, the combination of 30 µM kahweol acetate and 30 µM cafestol or 100 µM kahweol acetate administration reduced the expression of CCR2, CCR5, and CCR6 in both ACHN and Caki-1 cells (Fig. 5a,b). Moreover, with the emergence of immune checkpoint inhibitors as the prominent treatment method for renal cancer nowadays, the role of programmed death-ligand 1 (PD-L1) has also become a significant concern^7^. Accordingly, the combination of 30 µM kahweol acetate and 30 µM cafestol or 100 µM kahweol acetate administration reduced PD-L1 expression in both ACHN and Caki-1 cells (Fig. 5c,d). The aforementioned results indicated that kahweol acetate and cafestol may also affect the tumor microenvironment by inhibiting the immunological tolerance of renal cancer cells.

## Discussion

Recently, several studies have shown that kahweol or cafestol can regulate tumor cell activity and apoptosis-related proteins through multiple targets, individually contributing to the inhibition of renal cancer cell proliferation^27–30^. The present study examined the anti-cancer properties of coffee kahweol acetate and cafestol and their synergistic effects on two renal cancer cells, following our previous study focusing on prostate cancer cells^10^. Accordingly, our results showed that kahweol acetate and cafestol inhibited the proliferation and migration of both ACHN and Caki-1 cells, with their synergistic effects apparent at relatively low concentrations. Moreover, the combination of kahweol acetate and cafestol induced downregulation in not only anti-apoptotic proteins (Bcl-2 and Bcl-xL) but also EMT-related proteins. Inhibition of STAT3, Akt, and ERK signaling pathway, which play pivotal roles in tumor cell proliferation, invasion, and migration, had also been observed. Among apoptosis-related proteins, although the fragmentation of caspase-3 and PARP was not observed, Bax was increased by kahweol acetate and cafestol. Recently, Bax is reported to induce caspase-independent apoptosis^31^. The diterpenes may induce apoptosis through the caspase-independent apoptotic pathway: however, the role and the mechanism of this pathway in renal cell cancer should be further investigated. The aforementioned results therefore suggest that kahweol acetate and cafestol, which have been identified as potential anti-cancer agents, can directly suppress renal cancer cell activities.

**Figure 6:**
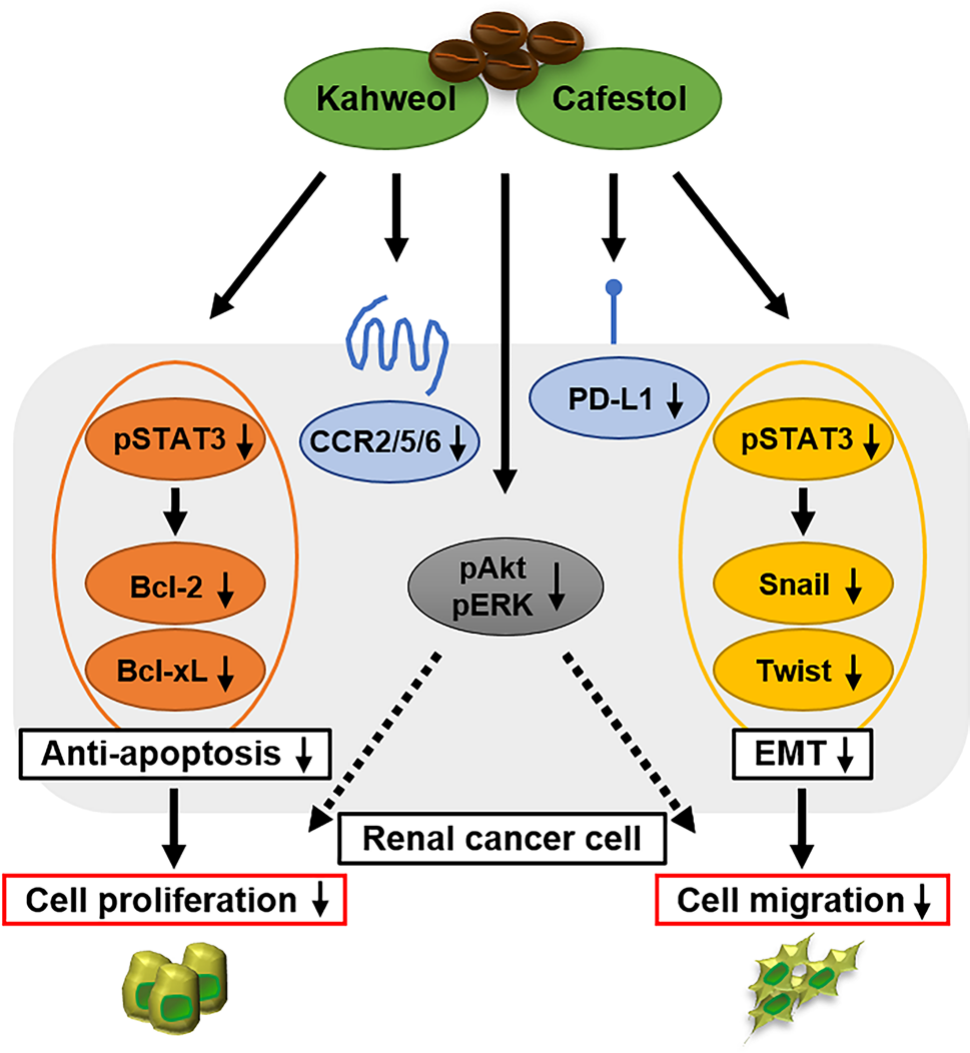
Schematic illustration of the anticancer mechanisms of kahweol acetate and cafestol. Kahweol acetate and cafestol inhibited cell proliferation and migration by inducing apoptosis and inhibiting epithelial–mesenchymal transition (orange and yellow colored molecules, respectively). Kahweol acetate and cafestol also inhibited CCR2/5/6, may potentially target the chemokine axis, and may affect the tumor immune environment by downregulating PD-L1 (blue colored molecules). Moreover, kahweol acetate and cafestol also inhibited the phosphorylation of Akt and ERK, which play central roles in tumor progression (gray colored molecules).

Immunotherapy, such as IFN-α and IL-2, had been the mainstream renal cancer therapy until the 1990s given the immunogenic property of renal cancer^32^. Recently, however, immune checkpoint inhibitors have become the prominent treatment method for renal cancer, with immune tolerance again becoming the main concern in the treatment of renal cancer^7^. Our previous studies had revealed that chemokines and their receptors were strong mediators of prostate and renal cancer progression with their effects on the tumor microenvironment^33–37^. Macrophage-like cells prepared from human monocytic leukemia cell line THP-1 promoted renal cancer cell migration, while CCL20, a specific ligand of CCR6, was more involved in macrophage-induced renal cancer cell migration than other cytokines^37^. Furthermore, AKT activation was involved in the promotion of renal cancer cell migration through the CCL20–CCR6 axis^37^. Some studies have already reported ERK activation is also induced by CCL20–CCR6 axis in cancer cells^38,39^. Therefore, it is interesting to note that kahweol acetate and cafestol treatment reduced CCR2, CCR5, and CCR6. The CCL2–CCR2 axis was reported to promote both cancer cell proliferation and migration by paracrine and by autocrine^40^. The CCL5–CCR5 axis also can arrange an immune-suppressive microenvironment, especially, BRCA1-associated protein 1-mutant clear cell renal cancer^41^. Moreover, studies have shown that PD-L1 expression was a negative prognostic factor and was associated with more advanced clinical features in patients with renal cancer^42,43^. Accordingly, our results showed that kahweol acetate and cafestol treatment also significantly inhibited PD-L1. T lymphocytes express PD-1 which is the receptor of PD-L1 and regulates the activity of T lymphocytes^44^. Therefore, inhibition of PD-L1 by kahweol acetate and cafestol treatment may increase the activity of T lymphocytes attacking renal cancer cells. An exploratory clinical study showed coffee consumption did not affect the number of T lymphocytes^45^. In addition, kahweol acetate and cafestol did not damage DNA of human peripheral lymphocytes^46^. Hence, although we did not clarify the direct effect of kahweol acetate and cafestol on T lymphocytes in this study, the diterpenes may not have significant effects on T lymphocytes. The aforementioned results thus indicate that kahweol acetate and cafestol can indirectly suppress renal cancer cell activities through by controlling the immunological tolerance of renal cancer cells. As shown in Fig. 6, kahweol acetate and cafestol exhibit multifunctional direct and indirect anti-tumor effects in renal cancer cells. Moreover, previous studies have suggested kahweol and cafestol could exhibit anti-angiogenic properties mainly by downregulating VEGF receptor-2, a primary mediator of the pro-angiogenic effect of VEGF^14^, with VEGF receptor inhibitor plus kahweol showing a synergistic effect in inducing renal cancer cell apoptosis^30^. Hence, the combination of kahweol acetate and cafestol may enhance the anti-cancer effects of conventional therapeutic agents, including tyrosine kinase inhibitors.

The current study revealed that kahweol acetate and cafestol may serve as not only therapeutic agents but also promising natural ingredients exhibiting anti-tumor effects against renal cancer cells. Although kahweol and cafestol are diterpenes included in unfiltered coffees, such as French press, espresso, and boiled coffees, concentrations of both diterpenes vary depending on the quality/blend and process of coffee preparation^47^. Studies have shown that 17.2 and 19.7 mg of kahweol and cafestol levels were present per cup (150 mL) of Arabica or Robusta coffee brewed using French press, respectively^12^. Assuming that an adult has a blood volume of 5000 mL and considering that approximately 70% of the consumed kahweol and cafestol can be absorbed in small intestine^11^, kahweol and cafestol concentrations may reach 30 µM each with three or four cups of coffee. Hence, achieving a concentration of 30 µM for both kahweol and cafestol, at which both exerted anti-cancer effects against renal cancer cells in our experiment, seems to be practicable.

In conclusion, the current study suggested that kahweol acetate and cafestol synergistically contributed to the inhibition of not only renal cancer proliferation and migration, by inducing apoptosis and inhibiting EMT, but also tumor microenvironment-related pathways, such as CCRs and PD-L1. Therefore, the combined use of kahweol acetate and cafestol may be a potentially effective therapeutic modality against renal cancer.

## Materials and methods

### Reagents and antibodies

Kahweol acetate (sc-228383A) and cafestol (sc-204663) were purchased from Santa Cruz Biotechnology (Dallas, TX, USA). The following antibodies were used during western blot analyses: mouse anti-Bcl-2 (15071S), rabbit anti-Bcl-xL (2764S), rabbit anti-STAT3 (8719S), rabbit anti-phospho-STAT3 (9131S), rabbit anti-Akt (9272S), rabbit anti-phospho-Akt (Ser473; 9271S), rabbit anti-ERK (9102S), rabbit anti-phospho-ERK (Thr202/Tyr204; 9101S), rabbit anti-PD-L1 (13684S), and horseradish peroxidase (HRP)-conjugated anti-rabbit IgG (7074S) antibodies from Cell Signaling Technology (Danvers, MA, USA); mouse anti-Snail (ab117866), mouse anti-Twist (ab175430), rabbit anti-CCR2 (ab155321), rabbit anti-CCR5 (ab32048), and rabbit anti-CCR6 (ab227036) from Abcam (Cambridge, MA, USA); mouse anti-GAPDH (NB300-221) from Novus Biologicals (Littleton, CO, USA); and HRP-conjugated anti-mouse IgG (1706516) antibody from Bio-Rad Laboratories (Hercules, CA, USA). Antibody information used in western blot analyses for Supplementary Figures is shown in Supplementary Figure S6.

### Cell culture

ACHN and Caki-1 human renal cancer cells, and HK-2 human normal kidney proximal tubular cells were purchased from the American Type Culture Collection (Manassas, VA, USA). Human renal cancer cell line cells and human kidney cell line cells were maintained in a growth medium containing RPMI-1640 (R8758; Sigma-Aldrich, St. Louis, MO, USA) and DMEM (D5796; Sigma-Aldrich), respectively, supplemented with 10% fetal bovine serum (FBS) and 1% penicillin/streptomycin (Invitrogen, Carlsbad, CA, USA) in a humidified incubator at 37 °C with 5% CO_2_.

### Cell proliferation assay

ACHN and Caki-1 cells were seeded in 12-well plates (5 × 10^4^ cells/well) with RPMI containing 10% FBS. Each was cell treated with or without predetermined concentrations of kahweol acetate and cafestol for 24 and 48 h. After the cells were harvested, cell numbers were counted using a hemocytometer.

### Cell migration assay

ACHN and Caki-1 cells (1 × 10^4^ cells/well) were seeded onto the upper chamber of transwell plates (cell culture inserts with 8.0-μm pore sizes for 24-well plates) with RPMI containing 0.1% FBS, while the lower compartment was filled with predetermined concentrations of kahweol acetate and cafestol in RPMI containing 10% FBS. Cells were then incubated for 12 h at 37 °C in a humidified incubator containing 5% CO_2_. Thereafter, the cells on the filter of cell culture inserts were fixed with 4% paraformaldehyde in phosphate-buffered saline (PBS) for 10 min. The cells on top of the filter were carefully removed with a cotton swab, while those on the back of the filter were stained with 0.1% crystal violet for 20 min. The stained filter was then microscopically photographed, after which migrated cells in two random fields were counted.

### Western blot analyses

Cell lysates were prepared using RIPA lysis buffer (FUJIFILM Wako Pure Chemical Corporation, Japan) containing 1% protease inhibitor cocktail and phosphatase inhibitor cocktail (Sigma-Aldrich). Soluble lysates (10–30 µg) were mixed with a lithium dodecyl sulfate sample buffer and sample reducing agent, both obtained from Thermo Fisher Scientific (Waltham, MA, USA), and separated through sodium dodecyl sulfate polyacrylamide gel electrophoresis. Separated proteins were then transferred to nitrocellulose membranes. The membranes were blocked with 1% gelatin and 0.05% Tween in Tris-buffered saline for 1 h at room temperature and then incubated overnight at 4 °C with primary antibody according to the manufacturer’s instructions. After washing, the membranes were incubated with HRP-conjugated anti-rabbit or anti-mouse secondary antibody for 1 h at room temperature. Protein bands were detected using the Super Signal West Femto maximum sensitivity substrate (Thermo Fisher Scientific). Full length images of cropped blots presented in Figs. 2, 3, 4, and 5 are shown in Supplementary Figure S7.

### Apoptosis assay

Apoptosis was detected using TUNEL assay with the DeadEndTM Fluorometric TUNEL System (Promega, Madison, WI, USA) as per the manufacturer's instructions. Briefly, ACHN and Caki-1 cells (3 × 10^4^ cells) were exposed to predetermined concentrations of kahweol acetate and cafestol for 12 h on sterile slide coverslips. The cells were then washed twice with PBS, fixed with 4% methanol-free paraformaldehyde for 25 min, washed twice with PBS, and permeabilized with 0.2% Triton X-100 for 20 min. After two more washes, each glass slide was covered with equilibration buffer for 10 min. Thereafter, the buffer was aspirated, and the glass slides were incubated with rTdT buffer at 37 °C for 1 h under shading. Chromosomal DNA was stained with 4′,6-diamidino-2-phenylindole (Sigma-Aldrich) for 15 min, after which the stained cells mounted on slides were examined using an OLYMPUS cellSens Standard (OLYMPUS, Japan).

### Combination index

Synergistic effect was evaluated using combination index (CI) analysis widely used for evaluating drug interactions in combination cancer chemotherapy. The CI is calculated using the following formula: CI = (D)_1_/(Dx)_1_ + (D)_2_/(Dx)_2_. In the denominator, (Dx)_1_ represents the concentration at which Drug 1 “alone” inhibits a system x%, while (Dx)_2_ represents the concentration at which Drug 2 “alone” inhibits a system x%. In the numerators, (D)_1_ and (D)_2_ represent the concentrations for Drug 1 and Drug 2 that (D)_1_ + (D)_2_ “in combination” inhibit a system x%. The CI-isobologram equation allows for the quantitative determination of drug interactions, where CI < 1, = 1, and > 1 indicate synergism, additive effect, and antagonism, respectively^13^.

### Statistical analyses

Protein expression was quantitatively analyzed through densitometry using ImageJ software (Bethesda, MD, USA). Graphs are presented as means ± standard error of mean (SEM). Statistical analysis was performed using GraphPad Prism version 6.07 (GraphPad Software, San Diego, CA, USA). Statistically significant differences were determined using one-way ANOVA followed by Tukey’s post hoc analysis, with *p* values < 0.05 indicating statistical significance.

## Supplementary Information

Supplementary information.

## Data Availability

All data generated or analyzed during this study are included in this published article.
