# Supplementary material for: Anti-proliferative and anti-migratory properties of coffee diterpenes kahweol acetate and cafestol in human renal cancer cells
Source: Sci Rep. 2021 Jan 12;11:675. doi: 10.1038/s41598-020-80302-4 (PMC7804192; doi:10.1038/s41598-020-80302-4)
Supplement: Supplementary file 1 — Supplementary information. [file 41598_2020_80302_MOESM1_ESM.pdf]

Anti-proliferative and anti-migratory properties of coffee diterpenes kahweol acetate and cafestol in human renal cancer cells

Tomoyuki Makino[1], Kouji Izumi[1,*], Kaoru Hiratsuka[1], Hiroshi Kano[1], Takafumi Shimada[1], Taito Nakano[1], Suguru Kadomoto[1], Renato Naito[1], Hiroaki Iwamoto[1], Hiroshi Yaegashi[1], Kazuyoshi Shigehara[1], Yoshifumi Kadono[1], Hiroki Nakata[2], Yohei Saito[3], Kyoko Nakagawa-Goto[3], Norihiko Sakai[4], Yasunori Iwata[4], Takashi Wada[4], and Atsushi Mizokami[1]

1. Department of Integrative Cancer Therapy and Urology, Kanazawa University Graduate School of Medical Science, Kanazawa, Japan
2. Department of Histology and Cell Biology, Kanazawa University Graduate School of Medical Science, Kanazawa, Japan
3. School of Pharmaceutical Sciences, College of Medical, Pharmaceutical and Health Science, Kanazawa University, Kanazawa, Japan
4. Department of Nephrology and Laboratory Medicine, Kanazawa University, Kanazawa, Japan.

***Correspondence to**:

Kouji Izumi, M.D., Ph.D., Department of Integrative Cancer Therapy and Urology, Kanazawa University Graduate School of Medical Science, 13-1 Takara-machi, Kanazawa, Ishikawa 920-8640, Japan.

Tel: +81-76-265-2393, Fax: +81-76-234-4263, E-mail: azuizu2003@yahoo.co.jp

Supplementary Figure S1. Anti-proliferative effects of kahweol acetate and cafestol on normal kidney cell; proximal tubular cells from normal adult human kidney (HK-2).

Cell proliferation assay

Human renal normal HK-2 cells were seeded in 12-well plates (5×10^4 cells/well) with DMEM containing 10% FBS. Each cells treated with or without pre-determined concentration of kahweol acetate and cafestol for 24 h. Cells were harvested and cell numbers were counted using a haemocytometer. Data are shown as means ± standard error of the mean (SEM) (n = 3). ****P < 0.0001.

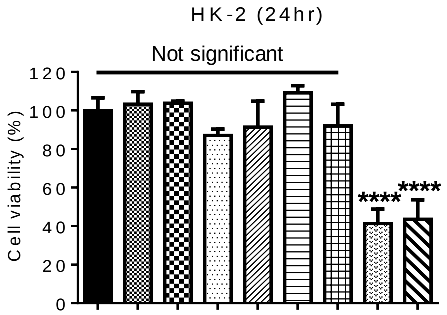

Kahweol	0	10	0	10	30	0	30	100	0
Cafestol	0	0	10	10	0	30	30	0	100

Supplementary Figure S2. Wound healing assay to additionally check the anti-migrative effect of kahweol acetate and cafestol on renal cancer cells.

Wound healing assay was performed using 12-well plates. ACHN and Caki-1 cells were let to grow until 90% confluence. A single wound was then scratched in the center of the cell monolayers with a 200-µl sterile plastic pipette tip. Subsequently, cells were incubated with treatment of kahweol acetate and cafestol in RPMI containing 10% FBS for another 12 h. The wound closure area was measured by fluorescence microscope (Keyence, Osaka, Japan). Bar in pictures, 500 µm. Data are shown as means ± SEM (n = 3). *P < 0.05; **P < 0.01. #Synergistic effects are observed.

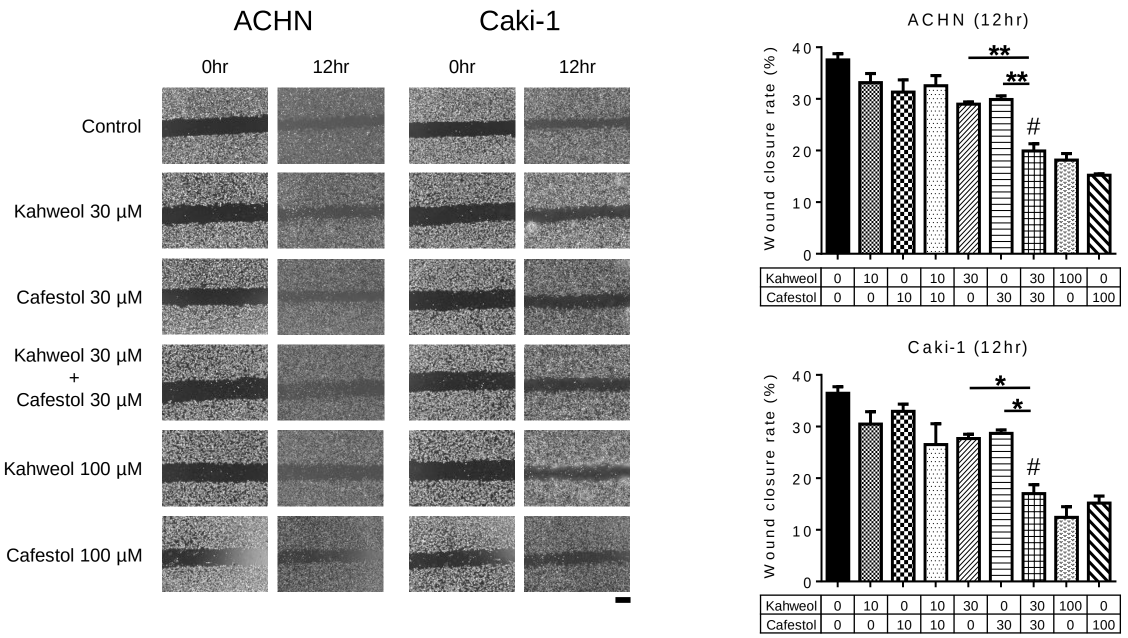

Supplementary Figure S3. Western blot analyses of pro-apoptotic protein, Bax.

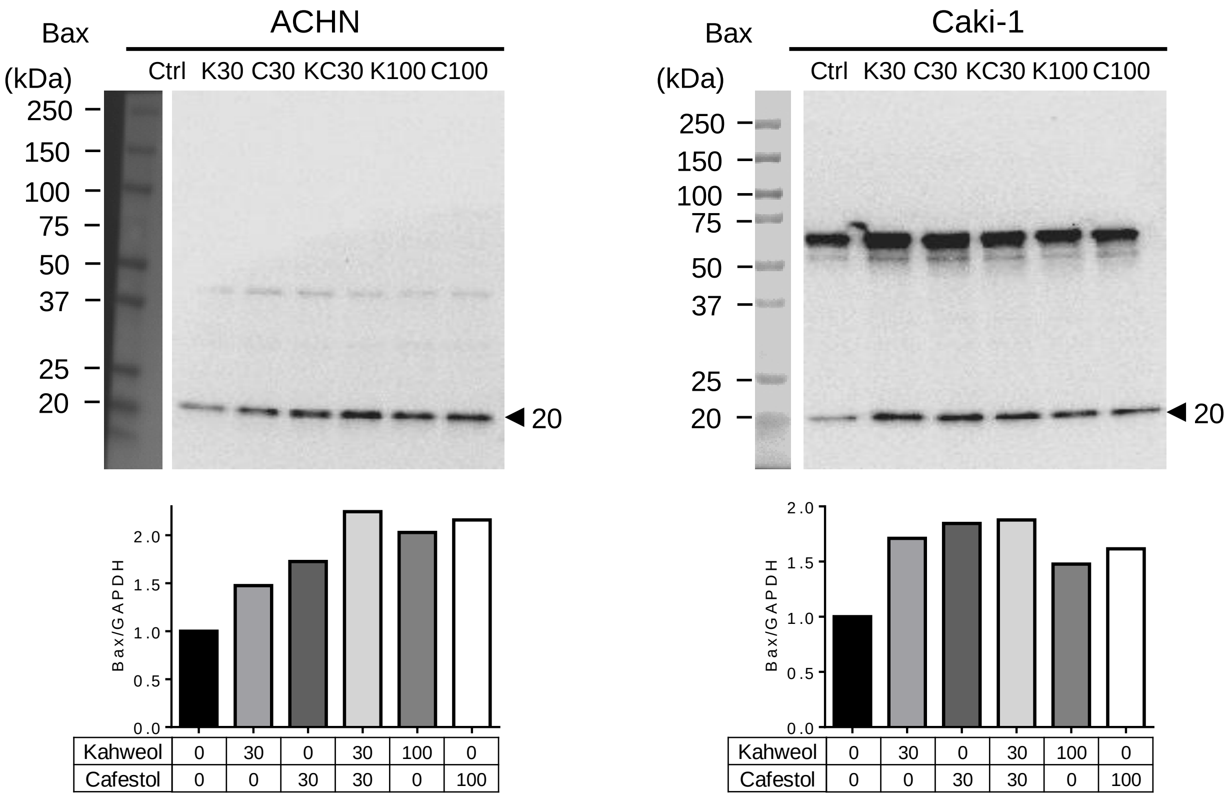

Supplementary Figure S4. Western blot analyses of the apoptosis-related proteins, cleaved caspase-3 and cleaved PARP.

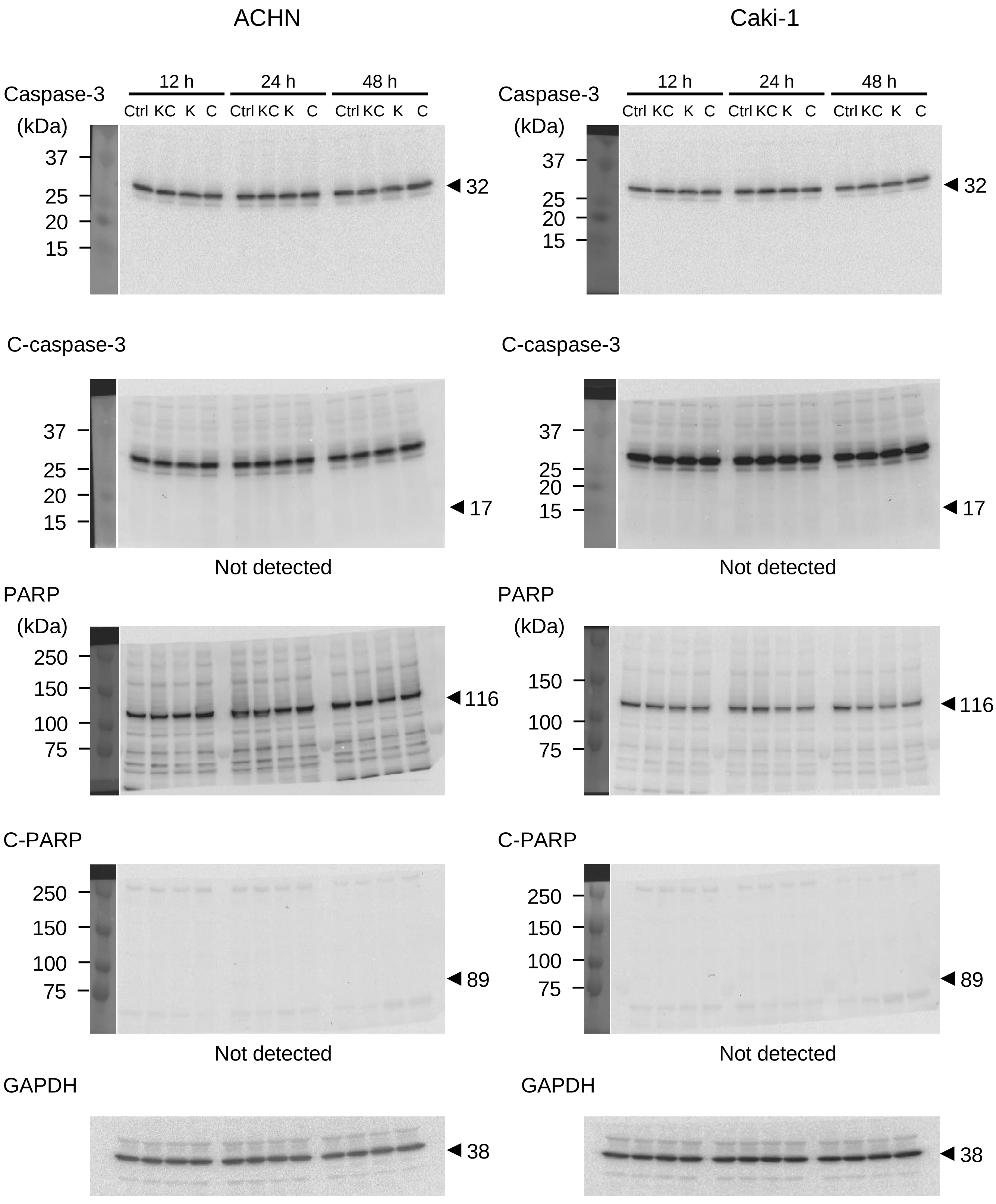

Ctrl: Control, KC: Kahweol 30 µM + Cafestol 30 µM, K: Kahweol 100 µM, C: Cafestol 100 µM

Supplementary Figure S5. Western blot analyses of the other EMT-related proteins.

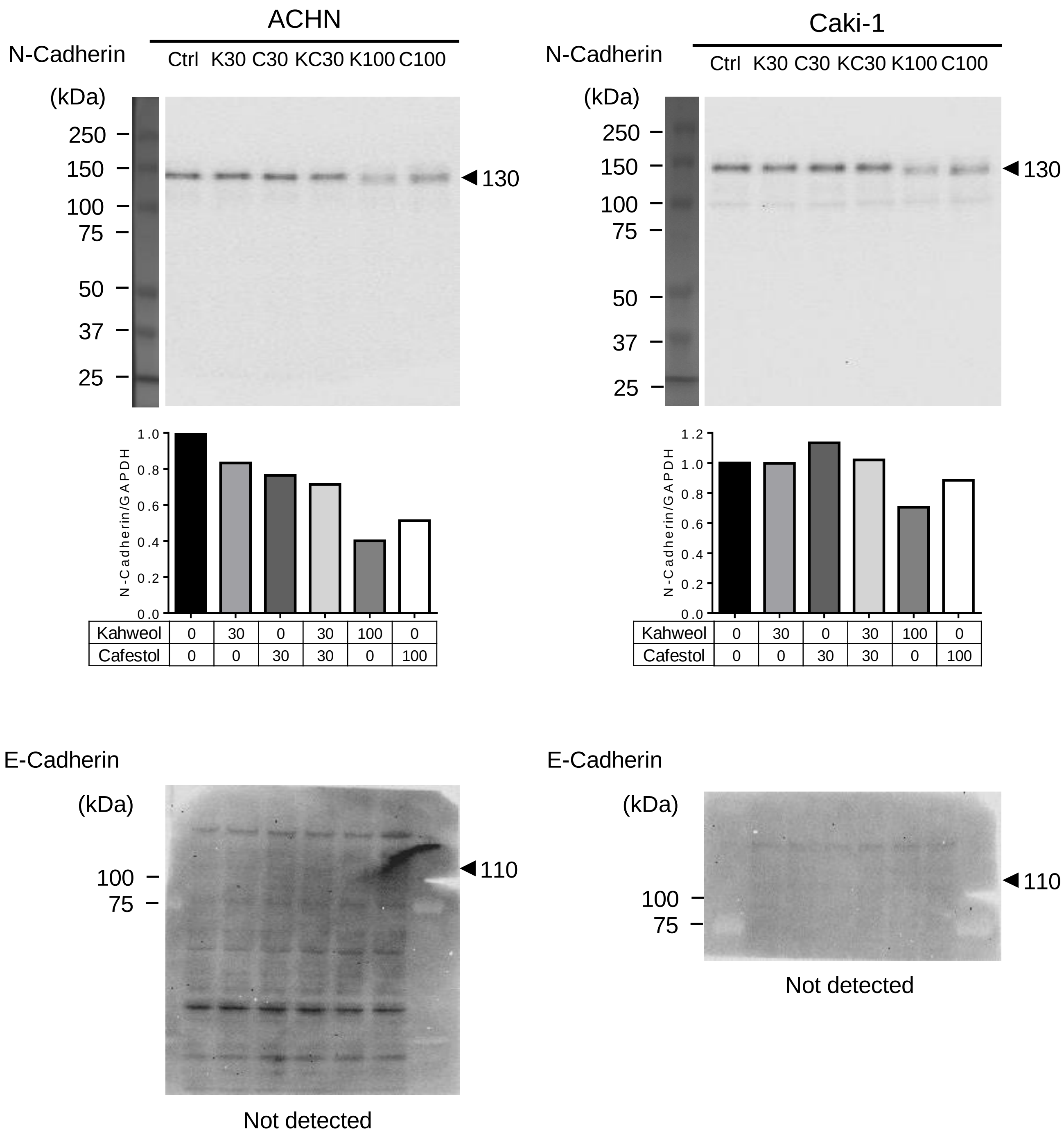

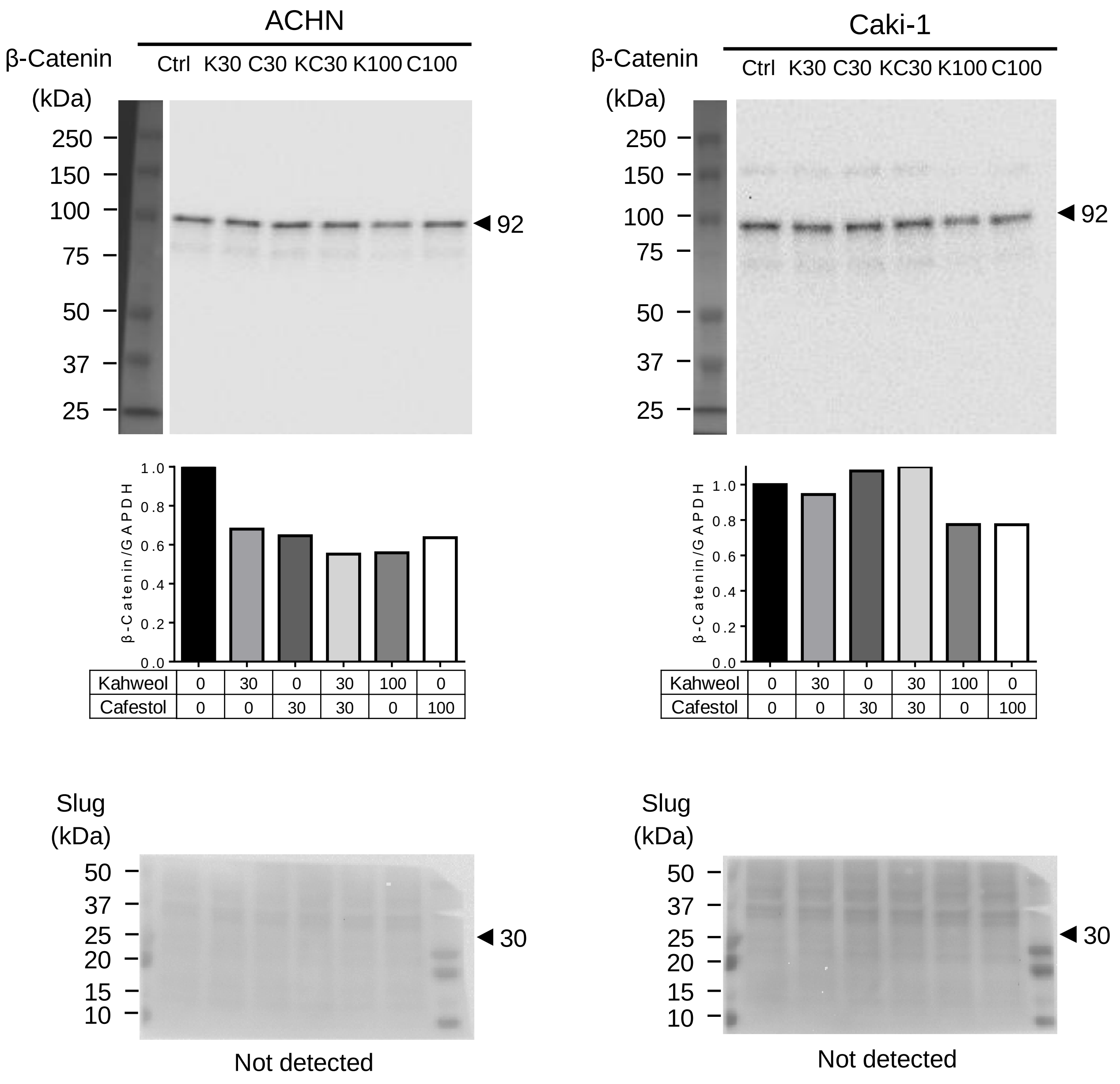
ACHN
β-Catenin
(kDa)
Ctrl K30 C30 KC30 K100 C100
250
150
100
75
50
37
25
92
β-Catenin/GAPDH
1.0
0.8
0.6
0.4
0.2
0.0
Kahweol 0 30 0 30 100 0
Cafestol 0 0 30 30 0 100
Caki-1
β-Catenin
(kDa)
Ctrl K30 C30 KC30 K100 C100
250
150
100
75
50
37
25
92
β-Catenin/GAPDH
1.0
0.8
0.6
0.4
0.2
0.0
Kahweol 0 30 0 30 100 0
Cafestol 0 0 30 30 0 100
Slug
(kDa)
50
37
25
20
15
10
30
Not detected
Slug
(kDa)
50
37
25
20
15
10
30
Not detected

Supplementary Figure S6. Antibody information used in western blot analyses for Supplementary Figures.

Antibodies	Predicted molecular weight (kDa)	Purchase source
rabbit anti-Caspase-3 (ab32351)	32	Abcam (Cambridge, MA, USA)
rabbit anti-Cleaved Caspase-3 (9661S)	17	Cell Signaling Technology (Danvers, MA, USA)
rabbit anti-PARP (9532S)	116	Cell Signaling Technology
rabbit anti-Cleaved PARP (5625S)	89	Cell Signaling Technology
rabbit anti-Bax (2772S)	20	Cell Signaling Technology
mouse anti-E-cadherin (ab1416)	110	Abcam
rabbit anti-N-cadherin (22018-1-AP)	130	Proteintech (Rosemont, IL, USA)
rabbit anti-β-Catenin (D10A8) (8480S)	92	Cell Signaling Technology
rabbit anti-Slug (C19G7) (9585S)	30	Cell Signaling Technology

Supplementary Figure S7. Full length images of cropped blots presented in Figures 2, 3, 4, and 5.

Figure 2

(c)

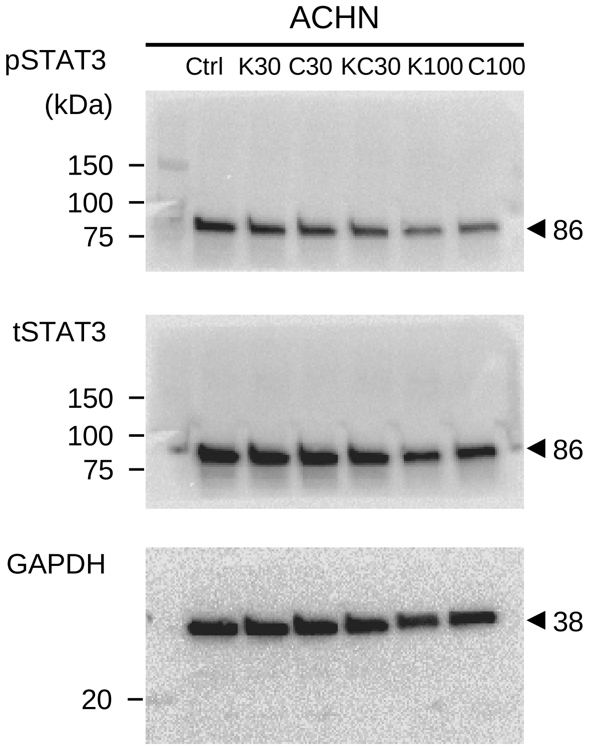

(d)

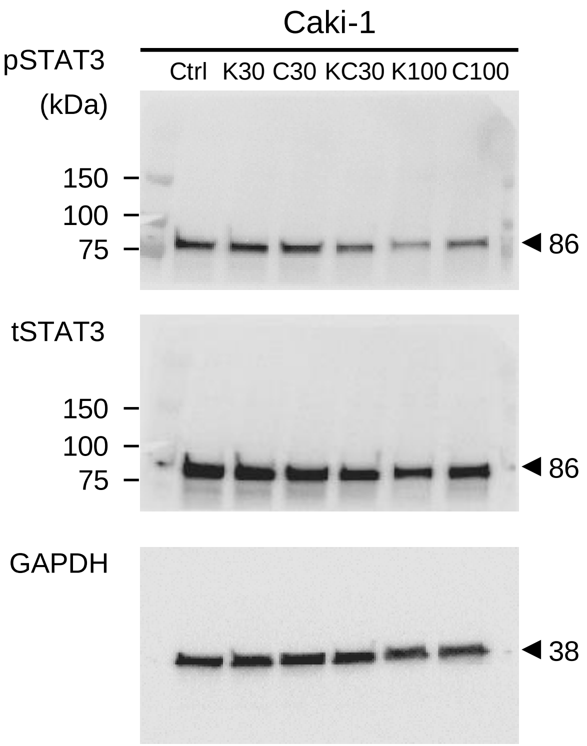

(e)

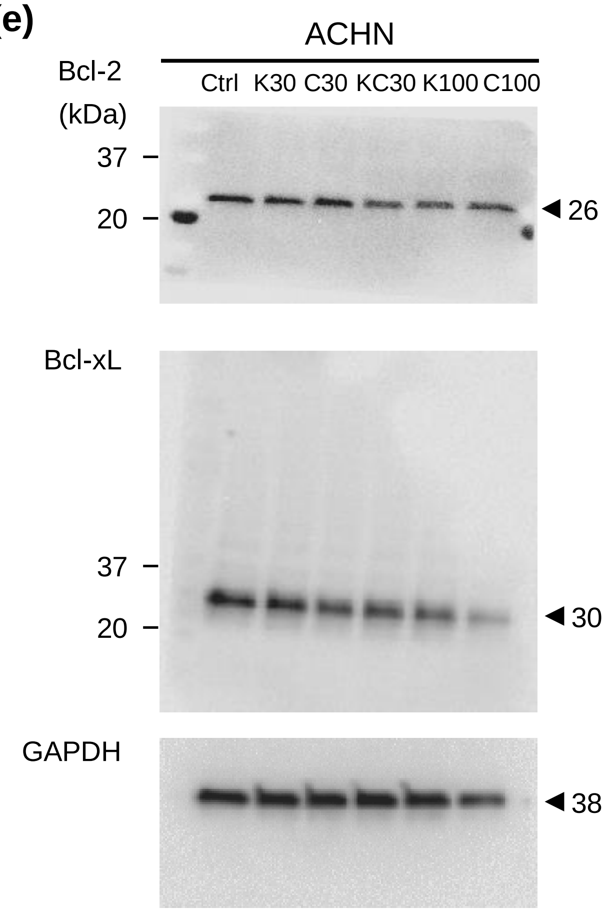

(f)

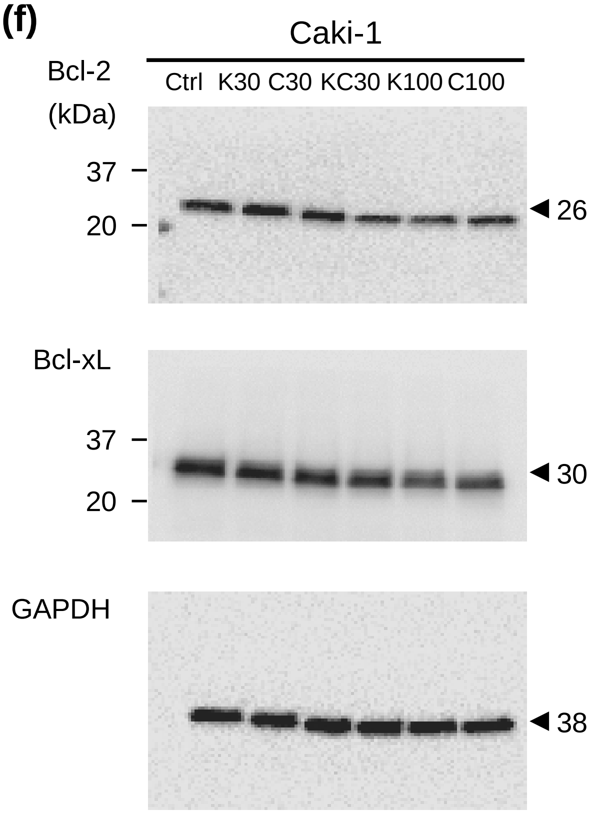

Ctrl: Control, K: Kahweol, C: Cafestol,
30: 30 μM, 100: 100 μM

Figure 3

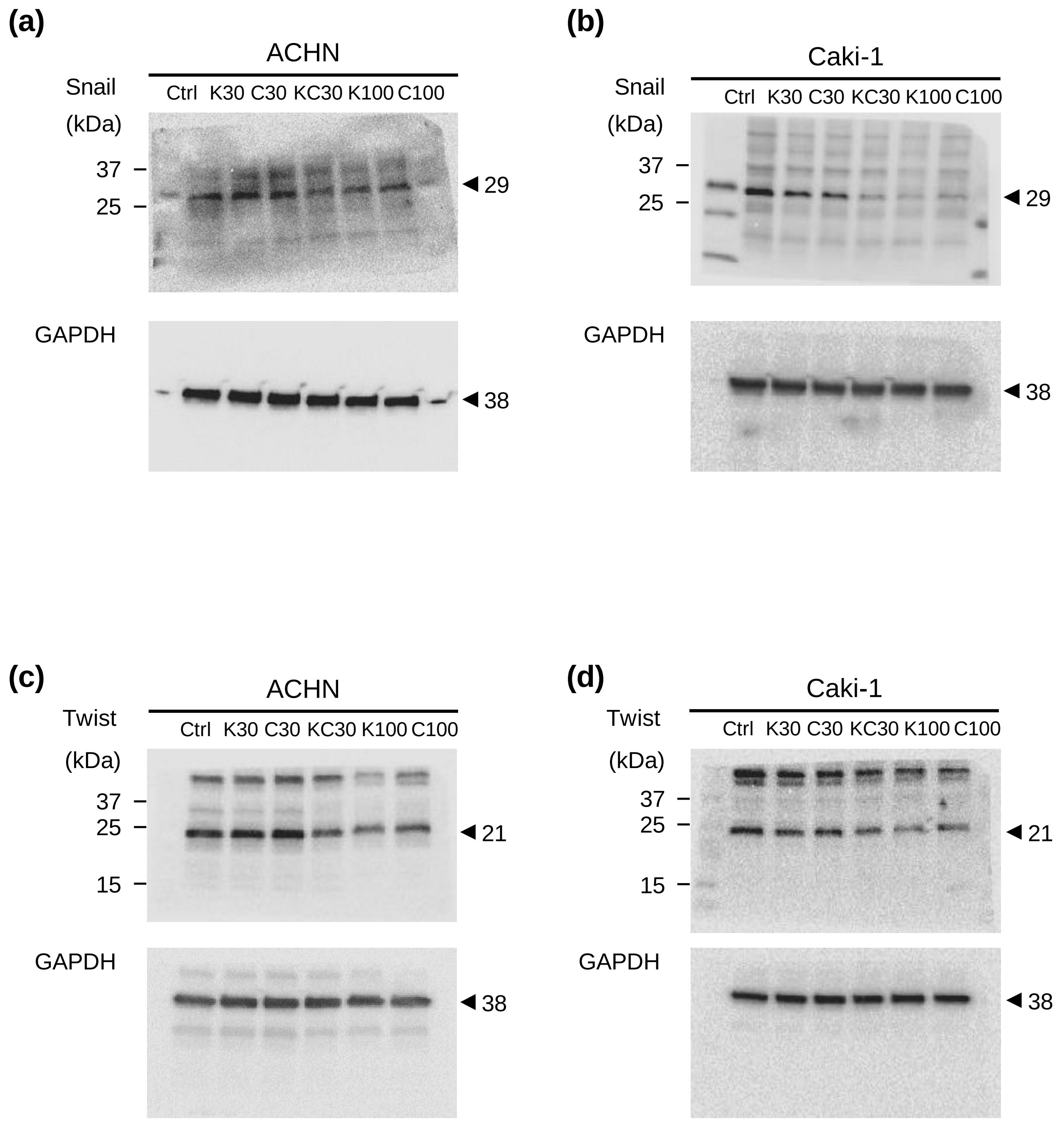
(a)
ACHN
Snail
Ctrl K30 C30 KC30 K100 C100
(kDa)
37
25
29
GAPDH
38
(b)
Caki-1
Snail
Ctrl K30 C30 KC30 K100 C100
(kDa)
37
25
29
GAPDH
38
(c)
ACHN
Twist
Ctrl K30 C30 KC30 K100 C100
(kDa)
37
25
15
21
GAPDH
38
(d)
Caki-1
Twist
Ctrl K30 C30 KC30 K100 C100
(kDa)
37
25
15
21
GAPDH
38

Figure 4

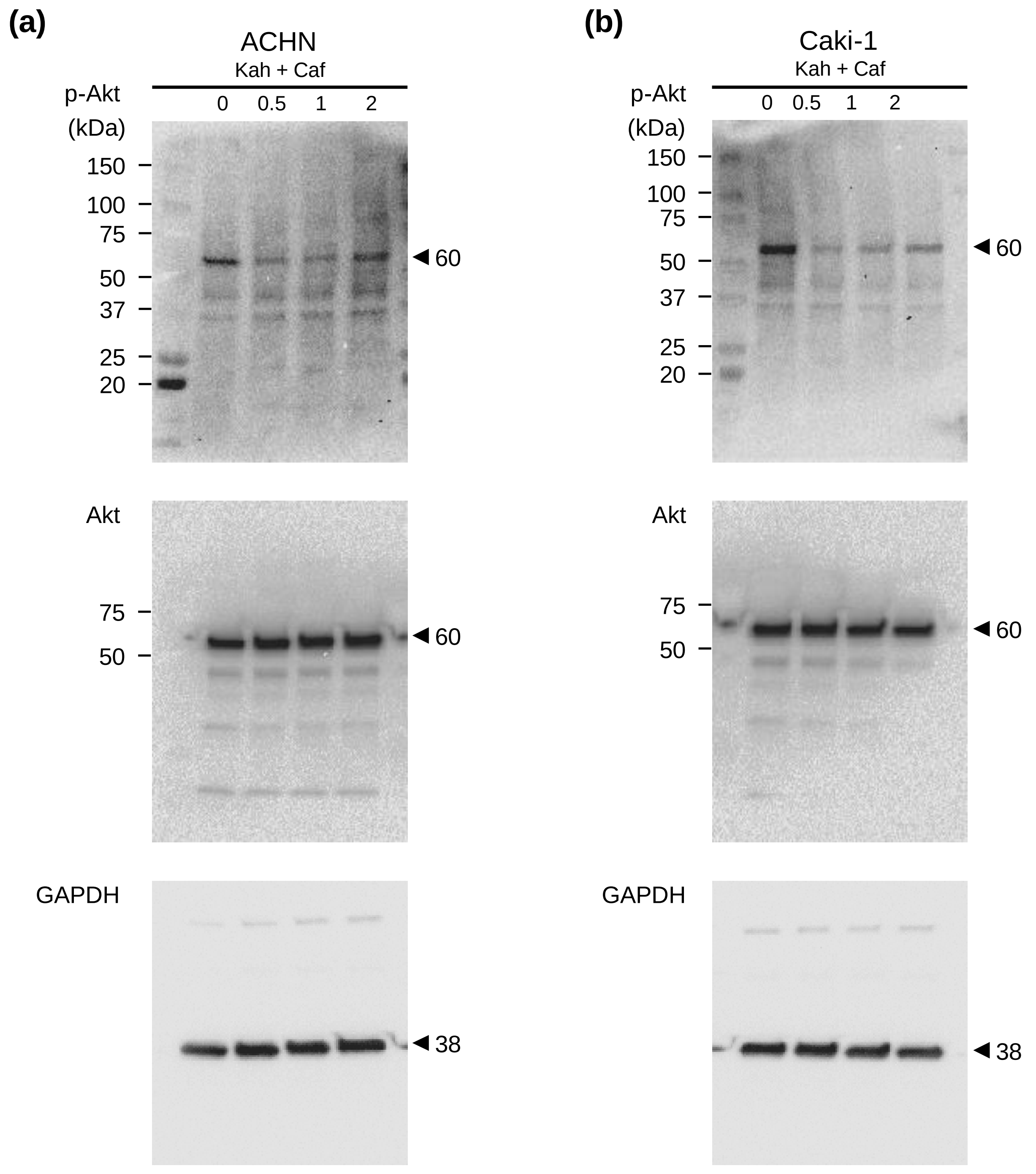
(a)
ACHN
Kah + Caf
p-Akt
0
0.5
1
2
(kDa)
150
100
75
50
37
25
20
60
Akt
75
50
60
GAPDH
38
(b)
Caki-1
Kah + Caf
p-Akt
0
0.5
1
2
(kDa)
150
100
75
50
37
25
20
60
Akt
75
50
60
GAPDH
38

Figure 4 continue

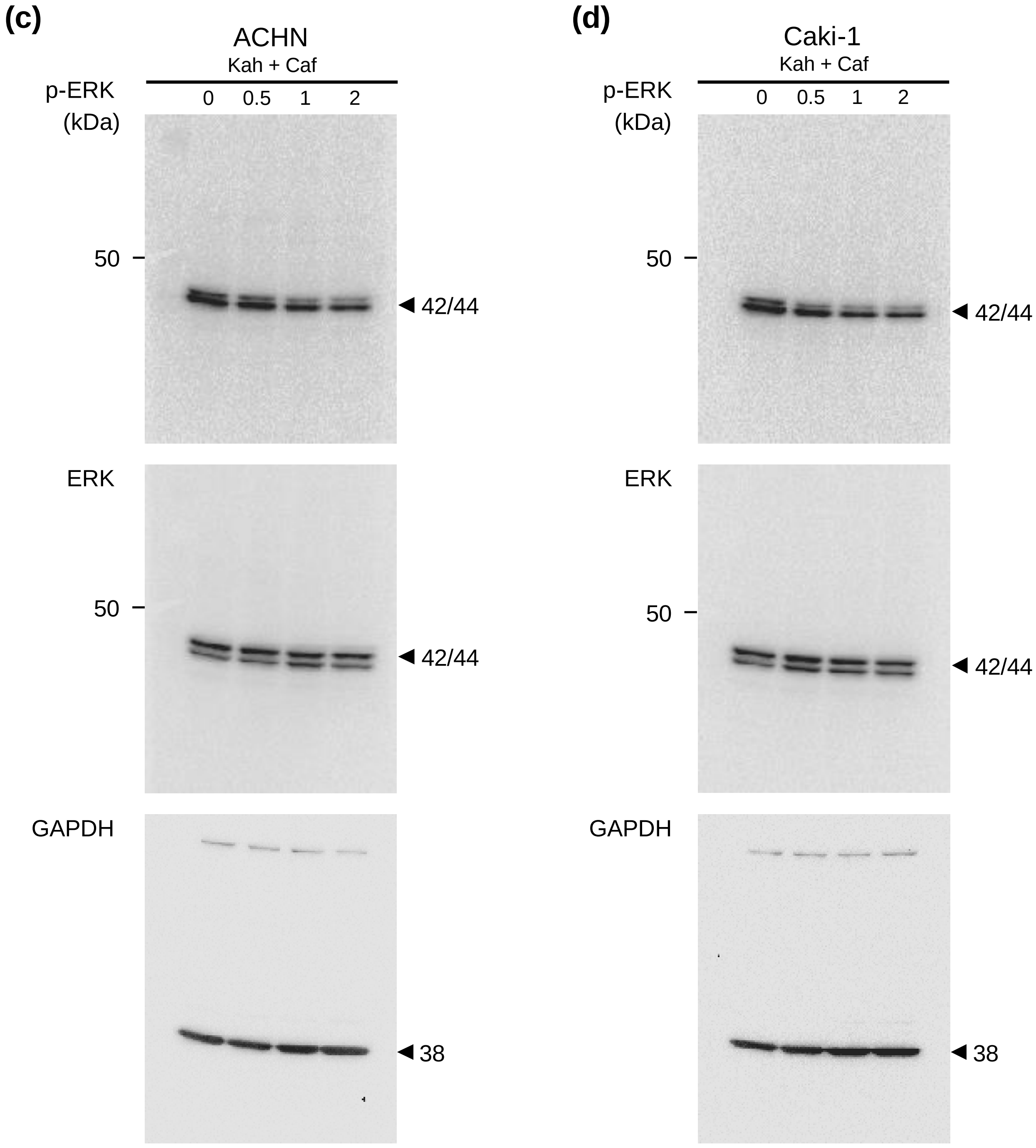

Figure 5

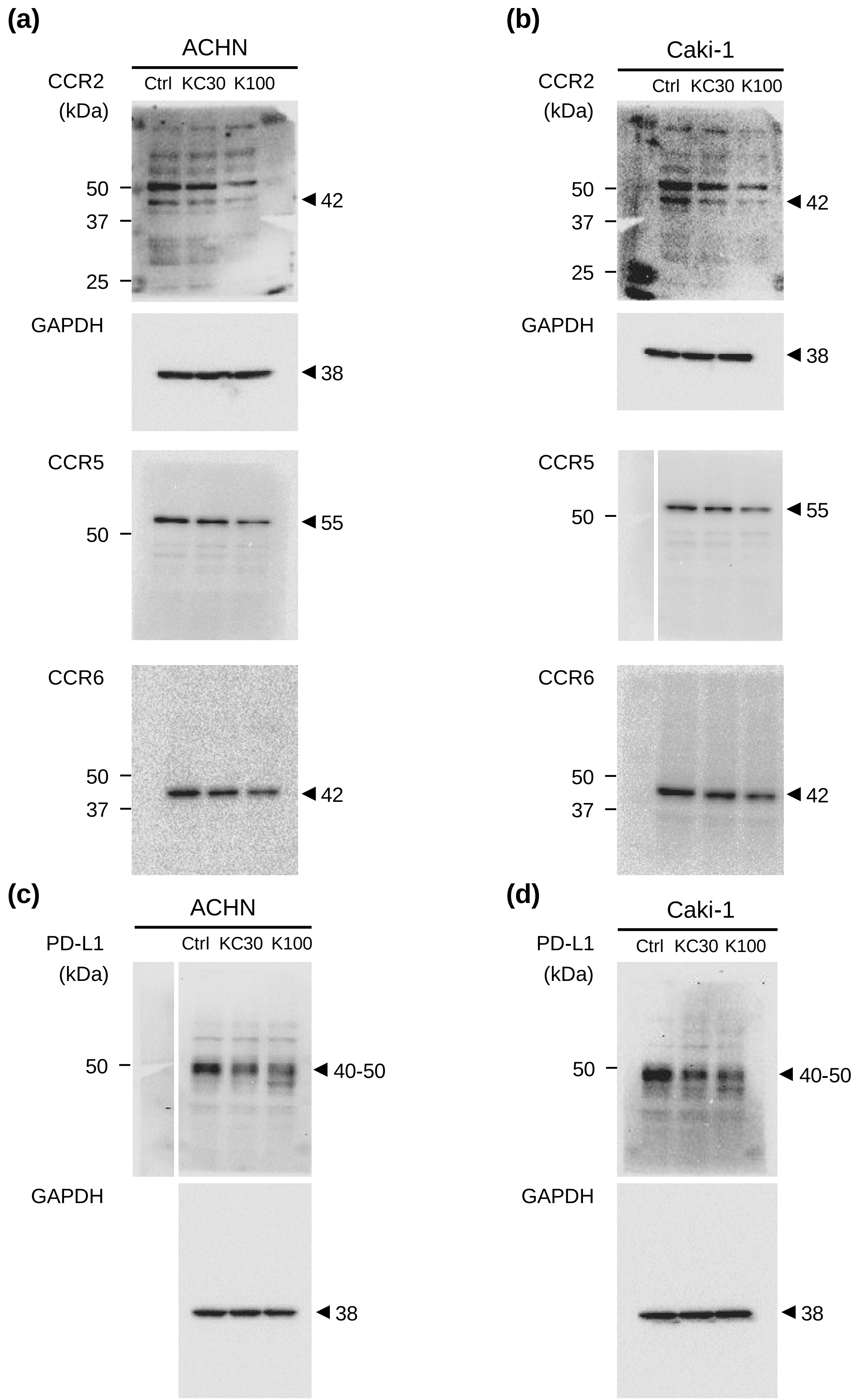
(a)
ACHN
CCR2
Ctrl KC30 K100
(kDa)
50
37
25
42
GAPDH
38
CCR5
50
55
CCR6
50
37
42
(b)
Caki-1
CCR2
Ctrl KC30 K100
(kDa)
50
37
25
42
GAPDH
38
CCR5
50
55
CCR6
50
37
42
(c)
ACHN
PD-L1
Ctrl KC30 K100
(kDa)
50
40-50
GAPDH
38
(d)
Caki-1
PD-L1
Ctrl KC30 K100
(kDa)
50
40-50
GAPDH
38
